# A Copartnership of Doctors, Homœopathy and Allopathy

**Published:** 1875-09

**Authors:** 


*A Copartnership of Doctors, Homœopathy and Allopathy.*—The *Boston Medical Journal*, in an editorial, August 12, 1875, makes certain observations upon the "Medical Profession in Michigan," from which we take the following:

"The struggle which has been maintained for so long a time between the regents of the State University and the homœopaths has terminated in the appointment of two homœopathic professors, the institution receiving a grant of six thousand dollars annually to defray the cost of instruction in homœopathy. It is stated that although these two chairs have been added to the medical faculty, the regulations have been so modified as to prevent this course from conflicting with the regular course of study, and the regents claim that the new professors are not members of the now existing department of medicine. That this is not the view which the State takes of the question, is shown by the fact that the attempt made by the regents, a few years ago, to establish a distinct homœopathic school, was ruled by the Supreme Court of the State to be not in compliance with the law. Moreover, Dr. Sager, the dean of the faculty, who has been connected with the University for nearly forty years, has felt himself called upon to resign, in the belief that the homœopathic branch has been practically engrafted upon the medical department. It is difficult to understand, under the present arrangement, why the other members of the faculty are not teachers in the homœopathic department. Practically this will be until two complete faculties are appointed. We regret to see it stated that the Michigan State Society refused to act upon a resolution condemning the course of the regents, who have, to say the least, placed the medical department of the University in a very equivocal position."

If colleges, or the theories that they teach and represent, can be allowed to form such copartnership, I can see no good reason why their graduates should not be allowed the same privilege. If a State Medical Society can endorse such a business and professional connection, it would certainly be inconsistent in that society to refuse admittance to homœopathic physicians. If the regular colleges can recognize such a mongrel enterprise, certainly their graduates, wherever they may be, should be allowed to mix and mingle, consult, trade and traffic in everything, and with everybody, regardless of creed, system, sex or color. The ethics should be interpreted the same in every State, and enforced to the letter, or it should be abolished. To scheme and plan with a purpose to dodge issues, for the benefit of personal friends, or to shirk responsibility, always seriously damages the individuals concerned, and, sooner or later, destroys the usefulness of the organizations involved. P.

